# A simple technique to position patients with bilateral above-knee amputations for operative fixation of intertrochanteric fractures of the femur: a case report

**DOI:** 10.1186/1752-1947-4-390

**Published:** 2010-11-30

**Authors:** Adeel Aqil, Aravind Desai, Asterios Dramis, Saqif Hossain

**Affiliations:** 1 Department of Orthopaedics, West Wales General Hospital, Carmarthen, UK; 2 Department of Orthopaedics, Rochdale Infirmary, Rochdale, UK; 3 Oxford Trauma Unit, John Radcliffe Hospital, Oxford, UK

## Abstract

**Introduction:**

Intertrochanteric fractures of the femur are common fractures in the elderly, and management includes operative fixation after patient positioning on the fracture table. Patients with bilateral above-knee amputations are challenging in terms of positioning on the table. We describe a simple technique to overcome this special problem.

**Case presentation:**

A 75-year-old wheelchair-bound Caucasian man with bilateral above-knee amputations presented to our hospital after a fall. Plain radiographs showed an intertrochanteric fracture of the femur, and operative fixation with a dynamic hip screw was planned. His positioning on the table posed a particular problem, and therefore we developed a technique to overcome this problem.

**Conclusion:**

Positioning of patients for fixation of intertrochanteric fractures of the femur poses a particular problem that can be solved by using our simple technique.

## Introduction

Fracture of the neck of the femur is a common indication for admission to trauma units [1]. Currently, the dynamic hip screw (DHS) is a common implant used in the fixation of extracapsular fractures of the proximal femur [2]. This involves positioning the patient on a fracture table and applying traction and rotation on the legs, after placing the feet in special boots fixed to the table. Therefore, positioning of patients with bilateral above-knee amputations is challenging, as their feet and part of their legs are missing.

A few methods have been described for patients with bilateral below-knee amputations undergoing fixation for intertrochanteric fractures [3,4]. We describe a simple technique for patients with above-knee amputations to overcome this problem.

## Case presentation

**Figure 1:**
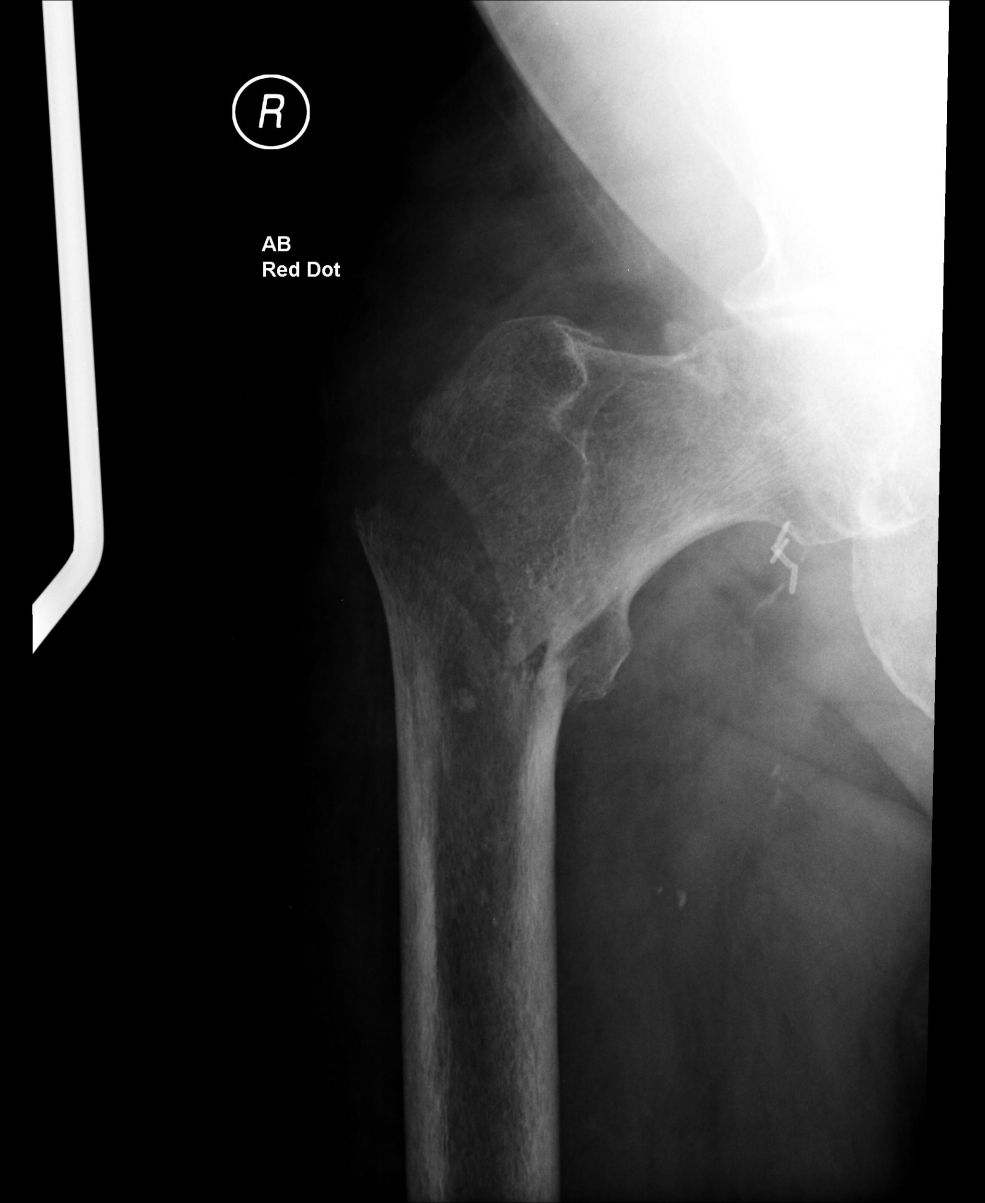
**Preoperative plain radiograph of the pelvis showing the intertrochanteric fracture of the right femur**.

A 75-year-old Caucasian man presented to our hospital after falling from his wheelchair. He complained of pain in his right hip, and plain radiographs showed a minimally displaced intertrochanteric fracture of the right femur (Figure 1). He had bilateral above-knee amputations for peripheral vascular disease but no prosthetic limbs, and therefore, he was wheelchair bound. A dynamic hip screw was planned, but we were faced with the dilemma of positioning the patient on the fracture table.

**Figure 2:**
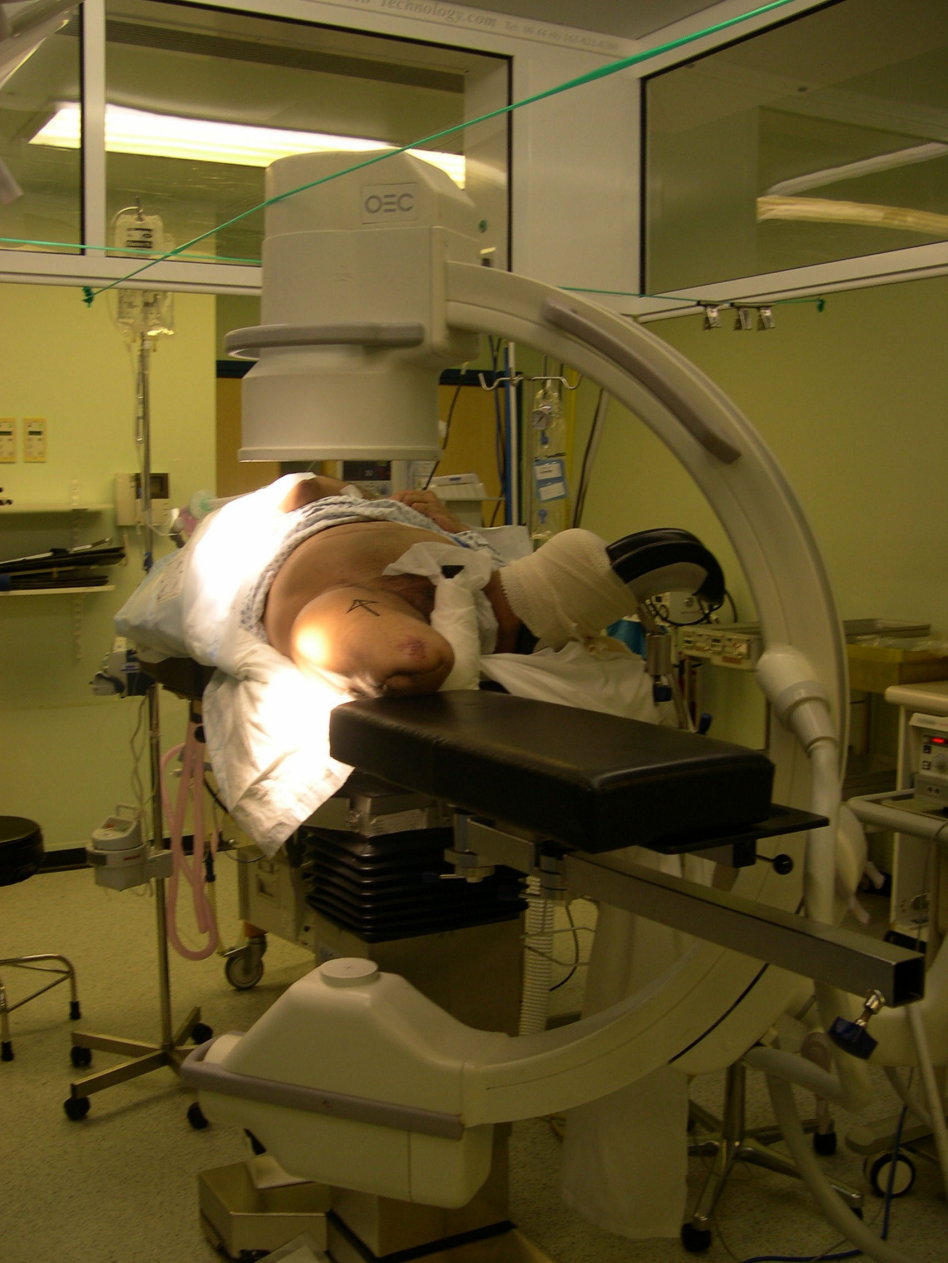
**Photograph of positioning of the patient on the fracture table with supports and the image intensifier adjusted for anteroposterior radiographs**.

**Figure 3:**
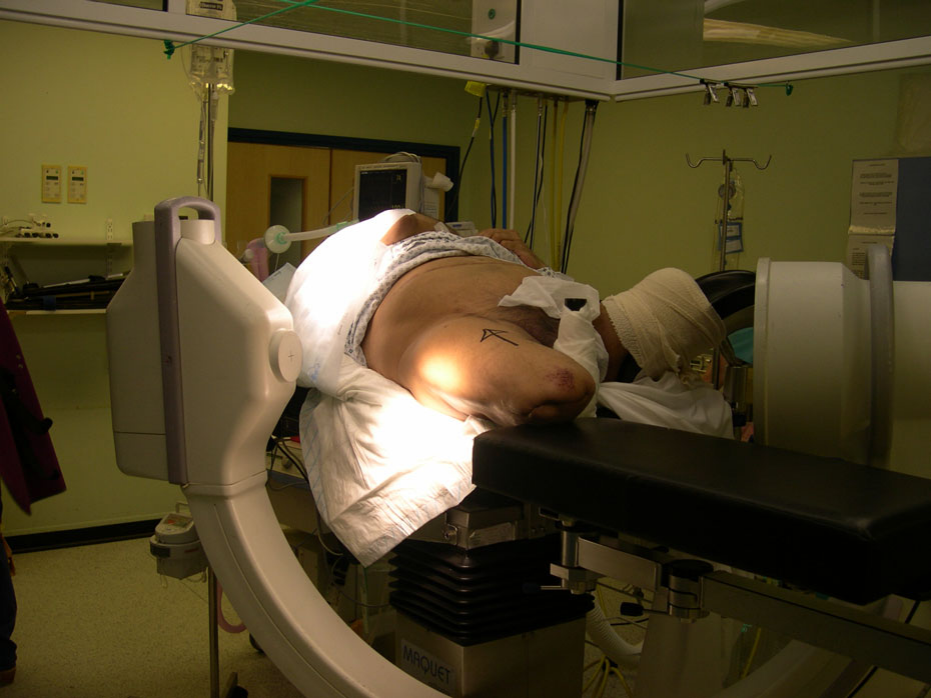
**Photograph of positioning of the patient on the fracture table with supports and the image intensifier adjusted for lateral radiographs**.

**Figure 4:**
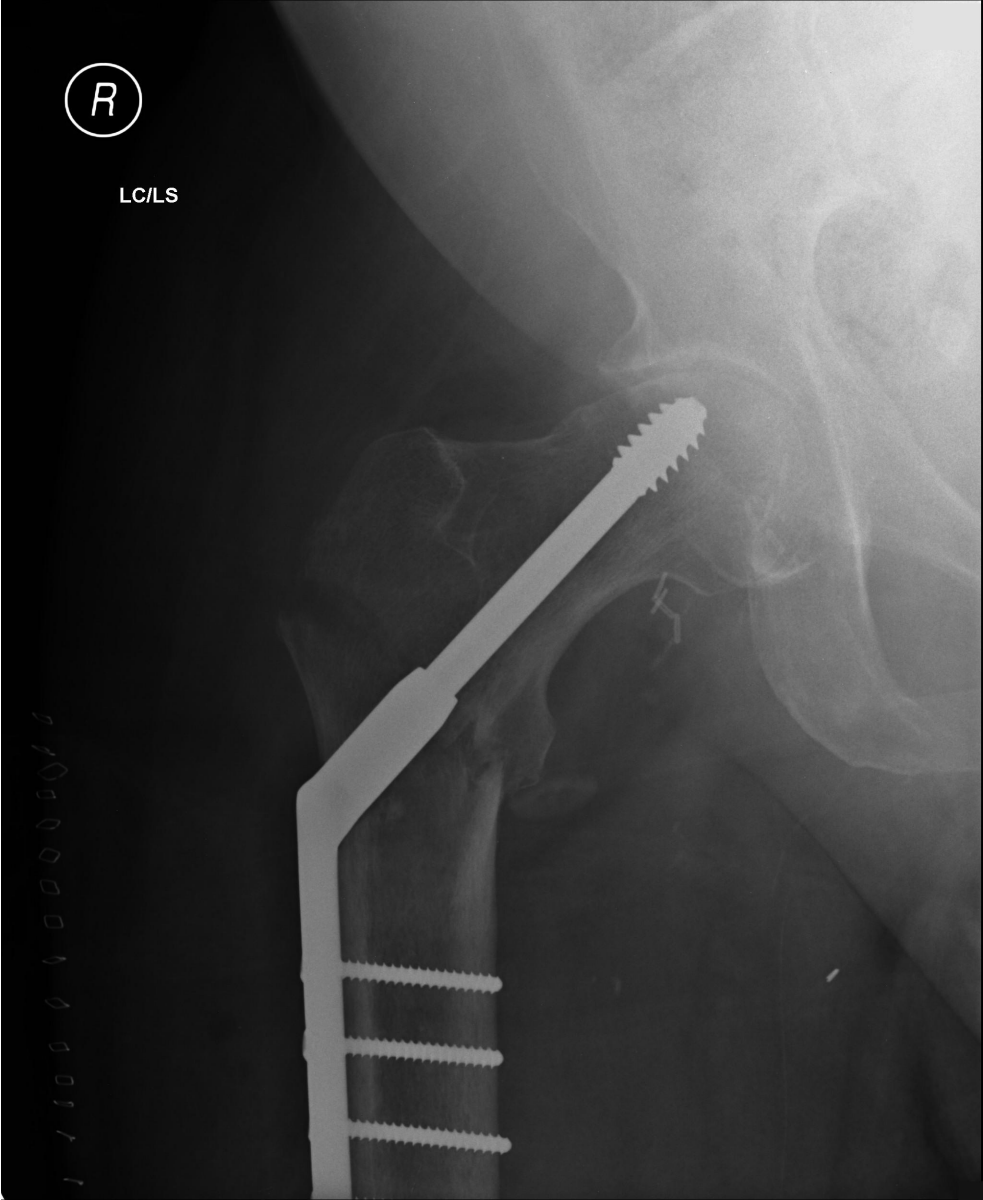
**Postoperative plain radiograph of the pelvis showing fracture fixation with a dynamic hip screw**.

The patient was placed supine on the radiolucent table, as in the standard procedure. The stump of the unaffected hip was bound firmly to a gutter support and placed in abduction and flexion, allowing good access for the image-intensifier arm. The stump on the fractured-hip side was placed on the thigh support of the fracture table without any traction component attached. Retaining the radiolucent thigh support allowed easy access for the image intensifier and visualization of the hip joint in both anterior-posterior (AP) and lateral views (Figures 2 and 3). Because the fracture was minimally displaced, *in situ *fixation of the fracture was carried out without any obstruction or difficulty under image-intensifier control (Figure 4). If further reduction were necessary, an attempt at closed reduction could have been carried out with direct traction along the thigh stump or by pin traction in the stump if needed, as attachment of any sort of traction device is not possible in such a short above-knee stump.

## Discussion

Patients with bilateral below-knee amputations and intertrochanteric fractures pose a special problem, as positioning them on the fracture table is difficult because of the absence of the feet and part of the legs.

The process of setting up the patient is important in achieving and maintaining fracture reduction while not causing skin injuries. Generally, the foot of the affected limb is put into a boot, and applying traction to this allows the fracture to be 'jacked out' and reduced. Internal rotation can then be applied if needed to achieve optimal fracture reduction. The unaffected limb is flexed at the hip and knee and strapped to allow the image intensifier to be moved into the groin region.

Closed reduction is preferred, but open-reduction techniques of such fractures have been described [3,4]. However, this conventional method could not be used in our patient, who had bilateral above-knee amputations with short stumps (10 cm on the left and 12 cm on the right). So far, one relevant operation technique was published in the literature regarding closed reduction and fixation of such a fracture in a patient with a unilateral below-knee amputation [5,6]. The authors describe flexing the knee and securing the padded stump to the inverted traction boot; the stump and knee act as a pseudo foot and ankle, thus allowing traction to be applied to the limb.

In the case of an above-knee amputation, even this technique cannot be applied.

## Conclusion

Fixation of intertrochanteric fractures of the femur in patients with above-knee amputations is a difficult problem for the surgeon in terms of positioning on the operating table. We describe a simple technique to overcome this problem and offer the surgeon an option to use when a similar case is encountered in trauma practice.

## Competing interests

The authors declare that they have no competing interests.

## Consent

Written informed consent was obtained from the patient for publication of this case report and any accompanying images. A copy of the written consent is available for review by the Editor-in-Chief of this journal.

## Authors' contributions

AA was involved in collecting patient details, reviewing the literature, and drafting the manuscript as the main author. AD was involved in reviewing the literature and proofreading the manuscript. AD* critically revised the manuscript for important intellectual content. SH was involved in the conception of the study and revising the manuscript. All authors read and approved the final manuscript.

## References

[B1] Parker M Johansen A Hip fracture: clinical review BMJ 2006 333 27 30 10.1136/bmj.333.7557.27 16809710 PMC1488757

[B2] Parker MJ Handoll HHG Extramedullary fixation implants and external fixators for extracapsular hip fractures in adults Cochrane Database Syst Rev 2006 1 CD000339 16437422 10.1002/14651858.CD000339.pub2

[B3] May JMB Chacha PB Displacement of trochanteric fractures and their influence on reduction J Bone Joint Surg (Br) 1968 50 318 323 5651338

[B4] Said GZ Farouk O Said HGZ An irreducible variant of intertrochanteric fractures: a technique for open reduction Injury 2005 36 871 874 10.1016/j.injury.2005.01.011 15949491

[B5] Al-Harthy A Abed R Campbell AC Manipulation of hip fracture in the below knee amputee Injury 1997 28 570 10.1016/S0020-1383(97)00118-6 9616404

[B6] Rethnam U Yesupalan RS Sohaib A Ratnam TK Hip fracture fixation in a patient with below-knee amputation presents a surgical dilemma: a case report J Med Case Rep 2008 2 296 10.1186/1752-1947-2-296 PMC2542403 18782438

